# Galectin-1 enhances TNFα-induced inflammatory responses in Sertoli cells through activation of MAPK signalling

**DOI:** 10.1038/s41598-018-22135-w

**Published:** 2018-02-27

**Authors:** Tao Lei, Sven Moos, Jörg Klug, Ferial Aslani, Sudhanshu Bhushan, Eva Wahle, Suada Fröhlich, Andreas Meinhardt, Monika Fijak

**Affiliations:** Department of Anatomy and Cell Biology, Justus-Liebig University, Giessen, Germany

## Abstract

Galectin-1 (Gal-1) is a pleiotropic lectin involved in the modulation of immune responses. Using a model of rat experimental autoimmune orchitis (EAO), we investigated the role of Gal-1 in testicular inflammation. EAO is characterized by leukocytic infiltrates in the interstitium, damage of spermatogenesis and production of inflammatory mediators like TNFα and MCP1 causing infertility. In normal rat testis Gal-1 was mainly expressed in Sertoli cells and germ cells. In the inflamed testis, Gal-1 expression was significantly downregulated most likely due to germ cell loss. Analyses of lectin binding and expression of glucosaminyl- and sialyltransferases indicated that the glycan composition on the cell surface of Sertoli and peritubular cells becomes less favourable for Gal-1 binding under inflammatory conditions. In primary Sertoli cells Gal-1 expression was found to be upregulated after TNFα challenge. Pretreatment with Gal-1 synergistically and specifically enhanced TNFα-induced expression of MCP1, IL-1α, IL-6 and TNFα in Sertoli cells. Combined stimulation of Sertoli cells with Gal-1 and TNFα enhanced the phosphorylation of MAP kinases as compared to TNFα or Gal-1 alone. Taken together, our data show that Gal-1 modulates inflammatory responses in Sertoli cells by enhancing the pro-inflammatory activity of TNFα via stimulation of MAPK signalling.

## Introduction

Infertility and subfertility affect 10–15% of couples and approximately 50% of cases are caused either by factors associated with the male alone or in combination with the female^1^. Infection and inflammation of the male genital tract are considered as one of the most important identifiable etiologies for male infertility^2,3^. Orchitis is characterized by the presence of inflammatory infiltrates in the testicular interstitium and associated disruption of seminiferous tubules, that can lead to partial or total impairment of spermatogenesis^4,5^. Acute epididymitis, orchitis or combined epidididymo-orchitis caused by infection show apparent clinical symptoms that can often be successfully treated with antibiotics and antiphlogistics^2^. Post- or non-infectious chronic orchitis is more hazardous because it is not associated with discomfort or pain, is difficult to diagnose and compromises testicular function^6–9^.

Experimental autoimmune orchitis (EAO) is a rodent model for studying organ-specific autoimmunity and chronic testicular inflammation that reproduces pathological changes also seen in some cases of human immunological infertility^10–12^. The initial phase of EAO involves the production of auto-antibodies against testicular antigens, increased migration and infiltration of leukocytes like macrophages, T lymphocytes and dendritic cells and elevated production of pro-inflammatory cytokines like TNFα and IL-6 or chemokines like MCP-1^13–15^. The chronic phase of the disease consists of granuloma formation, progressive apoptosis of germ cells, shrinkage of seminiferous tubules and decreased testicular weight^16–18^.

Galectins are a family of lectins characterized by a common structural fold and at least one conserved carbohydrate recognition domain (CRD) that recognizes β-galactose-containing glycoconjugates^19,20^. Gal-1 has a single CRD, requires reducing conditions to maintain its activities and is widely expressed in tissues of many vertebrates^21^. Through binding to specific glycan structures, Gal-1 is involved in a variety of physiologic and pathologic processes including pathogen recognition, selective induction of Th1 and Th17 apoptosis^22^, inhibition of T cell trafficking^23^, expansion of tolerogenic dendritic cells and regulatory T cells^24,25^, maintenance of maternal-fetal tolerance^26^, induction of pro-angiogenesis in anti-VEGF refractory tumors^27^ and suppression of an autoimmune pathology^28^. Gal-1 plays a role as the master regulator of clinically relevant inflammatory-response genes in osteoarthritic chondrocytes by stimulating NFκB-mediated inflammation^19^. Notably, the formation of galectin-glycan lattices decorating the cellular surface is a result of synchronized activities of glycan-modifying enzymes, glycosyltransferases and glycosidases^21^. Interestingly, Gal-1 expression in the testis exhibits a stage-specific pattern during the spermatogenic cycle, and immunostaining of Gal-1 in Sertoli cells is found mainly at stages X–II^29^. Moreover, Gal-1 is also expressed in human Sertoli cells^30,31^, but whether Gal-1 affects its immunoregulatory functions has not been elucidated yet.

In the present study, we investigated the expression of Gal-1 in rat EAO testis and the ability of Gal-1 to induce an inflammatory response in Sertoli cells. Moreover, the glycan profiles in EAO testes and TNFα challenged Sertoli as well as peritubular cells were investigated by using lectin binding assays.

## Results

### Due to germ cell loss expression of Gal-1 in EAO testis is decreased

**Figure 1:**
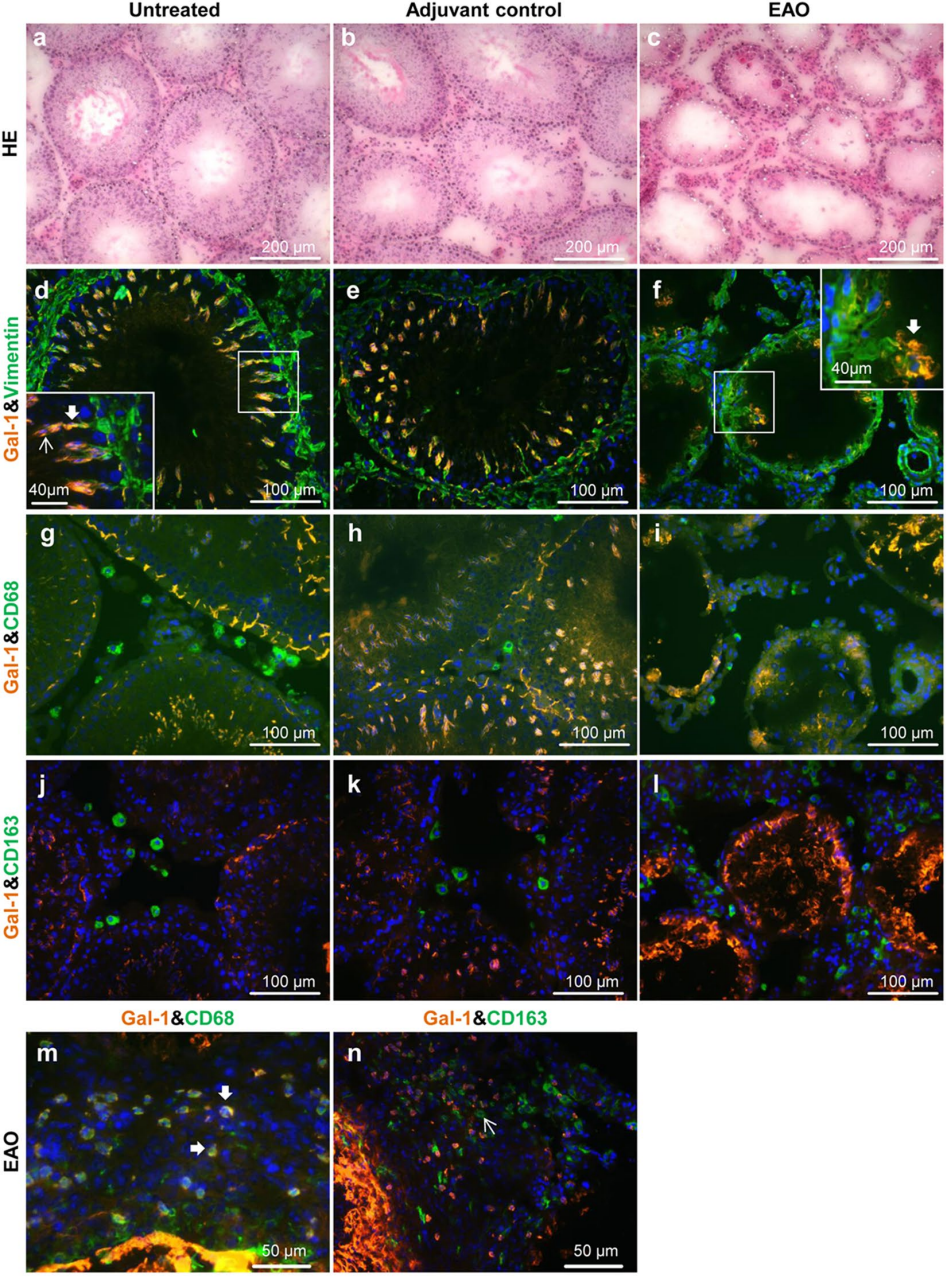
In normal rat testes Gal-1 is expressed mainly in Sertoli cells and germ cells but not in macrophages. Hematoxylin-eosin (HE) staining in cryostat sections from normal (**a**), adjuvant control (**b**) and EAO (**c**) rat testes. Localization of Gal-1 (Alexa 546, orange) in normal (**d**,**g**,**j**), adjuvant control (**e**,**h**,**k**) and EAO (**f**,**i**,**l**,**m**,**n**) testis. Vimentin (Alexa 488, green) was used as a marker of Sertoli cells (**d**,**e**,**f**). Insets show Gal-1 (Alexa 546, orange) stained in germ cells (thin arrow) and Sertoli cells (thick arrow) (**d**,**f**). Staining of Gal-1 and CD68 (Alexa 488, green) or CD163 (Alexa 488, green) in the region of granulomas (**m**,**n**). Testicular macrophages were stained with CD68 and CD163 antibodies. Gal-1 was expressed in some CD68 macrophages (**m**) found around granulomas (thick arrow), but not in CD163 macrophages (**n**) (thin arrow).

As described earlier^11,13^ histopathological changes in EAO testis include strong infiltration of the interstitium by leukocytes and loss of the germinal epithelium (Fig. 1c) that is accompanied by a reduced testicular weight^11^. Testes from untreated and adjuvant controls showed a completely normal morphology (Fig. 1a,b).

**Figure 2:**
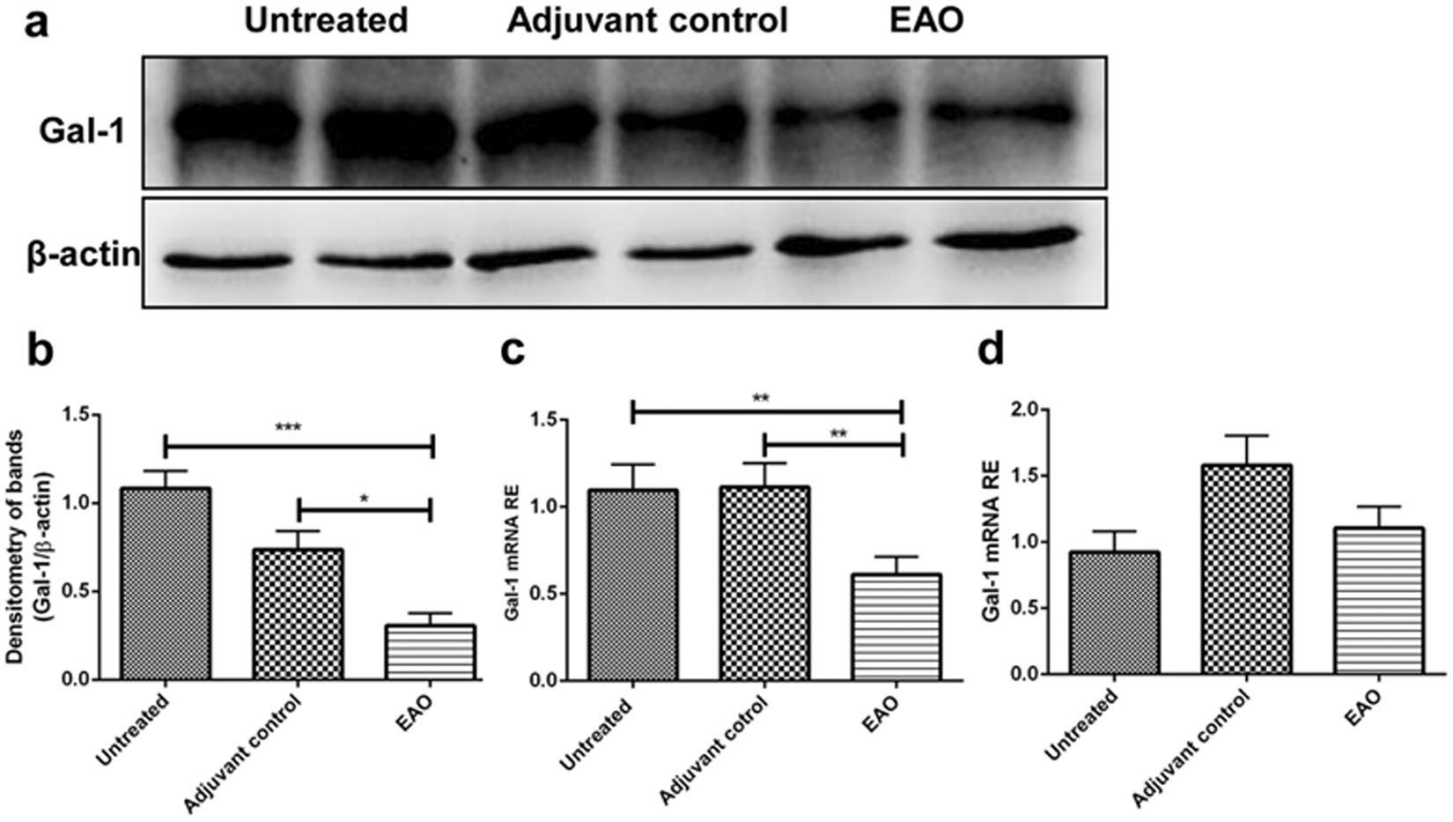
Changes in the expression of Gal-1 in EAO testes are due to germ cell loss. Western blot (**a**) and densitometric (**b**) analysis of Gal-1 expression in testes from untreated, adjuvant control and EAO animals. Gal-1 mRNA relative expression (RE) was normalized to three house keeping genes (β-actin, Hprt and 18 s rRNA) (**c**) or Sertoli cell marker Sox9 (**d**). The blots were cropped and the full-length blots are presented in the supplementary data (Supplementary Fig. 1); (n = 5, *P < 0.05, **P < 0.01, ***P < 0.001).

In order to investigate testicular expression and localization of Gal-1 in the EAO model, rat testes from untreated, adjuvant control and EAO mice were processed for immunofluorescence staining, Western blot and qRT-PCR analyses. Immunofluorescence staining revealed that in normal testis Gal-1 was localized in seminiferous tubules, mainly in the cytoplasm of Sertoli cells as co-localization with vimentin showed, as well as in germ cells (Fig. 1d–f). In normal testis Gal-1 was detected in the basal and apical cytoplasm of Sertoli cells (Fig. 1d), but not in CD68/CD163 macrophages (Fig. 1g–l). Interestingly, in inflamed testis Gal-1 was also detected in a few CD68+ macrophages located in the vicinity of granulomas (Fig. 1m). Of note, the expression of Gal-1 was not observed in CD163+ macrophages both in normal and EAO testes (Fig. 1j–l,n). Gal-1 protein levels in EAO testes were downregulated as compared to normal and adjuvant control testes (Fig. 2a,b, Supplementary Fig. 1). Similarly, relative expression of Gal-1 mRNA was also reduced in inflamed testes (Fig. 2c). Because the ratio of testicular cell types is changed in EAO testis due to the loss of germ cells and infiltration of immune cells, the relative expression of Gal-1 mRNA was normalized to the Sertoli cell specific transcript Sox9 (Fig. 2d). These data indicate that the mRNA expression of endogenous Gal-1 in Sertoli cells was not changed in EAO testis as compared to control testis at the investigated time point.

### Increase of St6gal1 mRNA expression and terminal α-2-6-sialylation in EAO testis

**Figure 3:**
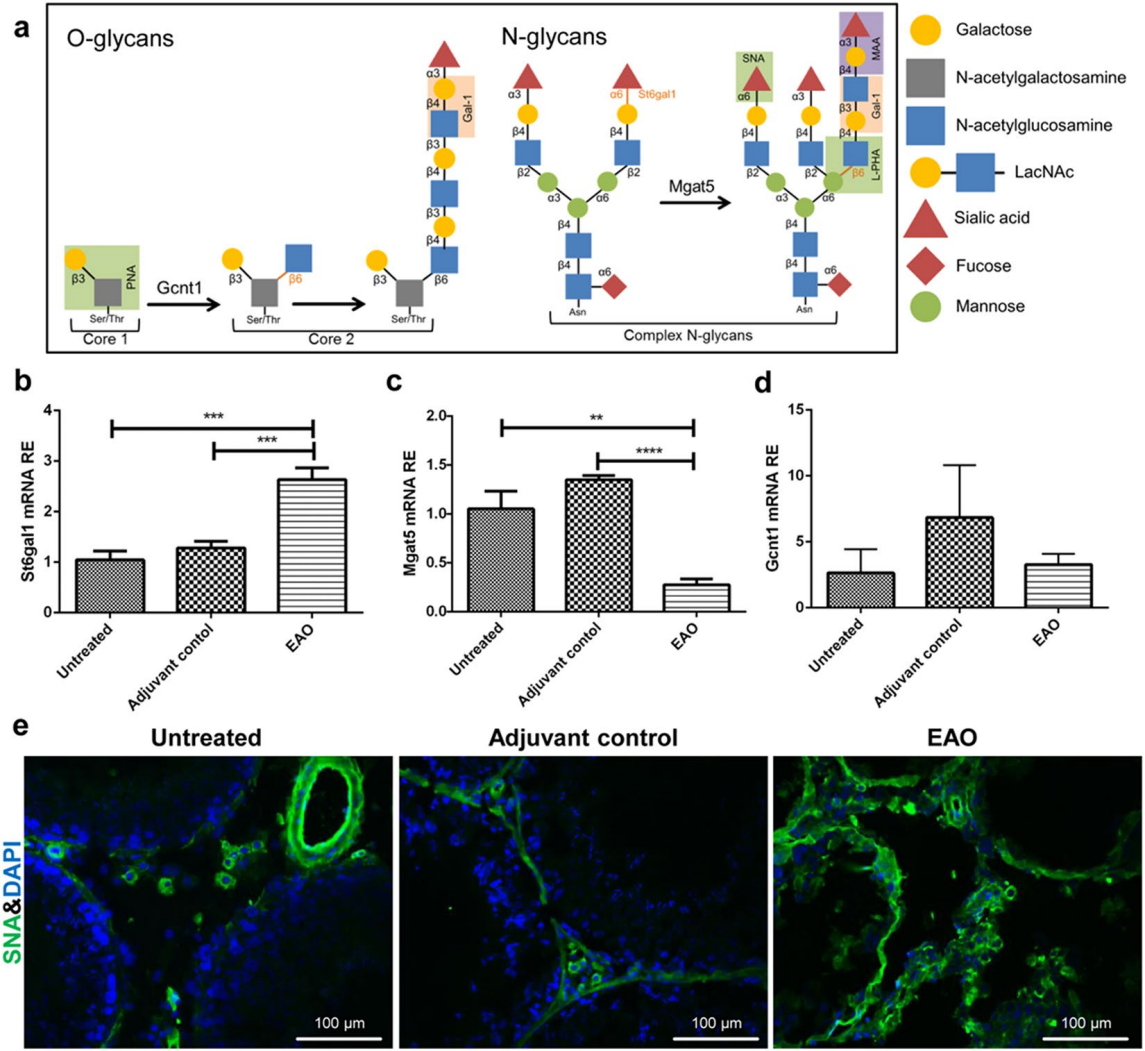
Expression analysis of transferases involved in glycan biosynthesis and lectin-FITC binding in EAO testes. (**a**) Schematic representation of N- and O-glycan biosynthesis. St6gal1 (**b**), Mgat5 (**c**) and Gcnt1 (**d**) relative mRNA expression was normalized to β-actin, Hprt and 18s rRNA (n = 5). (**e**) SNA-FITC binds stronger to testicular sections from EAO rats; (**P < 0.01, ***P < 0.001).

Gal-1 binds to N-acetyllactosamine (LacNAc) present on branches of N- and O-glycans on the cell surface which are synthesized by the synchronized activity of glycosyltransferases^21^. There are three important post-translational mechanisms to form Gal-1 binding sites including: (a) activity of core 2 glucosaminyl (N-acetyl) transferase 1 (Gcnt1) for synthesis of core 2 O-glycans, which are the backbone of Gal-1 ligands, (b) suppression of ST6 beta-galactoside α-2-6-sialyltransferase 1 (St6gal1) activity, that abrogates Gal-1 binding to some terminal N-acetylglucosamines by adding α-2-6-sialic acid and (c) branching of N-glycans by mannosyl (α-1,3-)-glycoprotein ß-1,2-N-acetylglucosaminyltransferases (Mgat) like Mgat5 (Fig. 3a)^21^. Our results show that the level of St6gal1 mRNA in EAO testes was upregulated (Fig. 3b). At the same time, binding of SNA, that recognizes terminal α-2-6 sialic acid residues (red triangles in Fig. 3a), was increased as compared to untreated and adjuvant control testis (Fig. 3e). In contrast, Mgat5 mRNA expression was downregulated and, consequently, binding of *Phaseolus vulgaris* agglutinin (L-PHA), that recognizes ß-5-ß-4-N-acetyl-glucosamine (blue squares in Fig. 3a), was reduced in EAO testis (Fig. 3c and Supplementary Fig. 2). These data indicate that α-2-6-sialylation of O- and N-glycans is increased in inflamed testis. Notably, expression of Gcnt1 mRNA was unchanged (Fig. 3d), whereas binding of peanut agglutinin (PNA), that recognizes asialo-galactose ß-1-3-N-acetylgalactosamine (core-1) in O-glycans, was decreased in EAO testis (Supplementary Fig. 2). However, we did not observe any significant change in the binding of *Maackia amurensis* agglutinin (MAA), that is recognizing NeuNAcα (2-3) Galβ (1-4) GlcNAc/Glc, in EAO testis (Supplementary Fig. 2).

### The binding of SNA to Sertoli and peritubular cells is increased after TNFα stimulation, whereas binding of L-PHA is decreased

**Figure 4:**
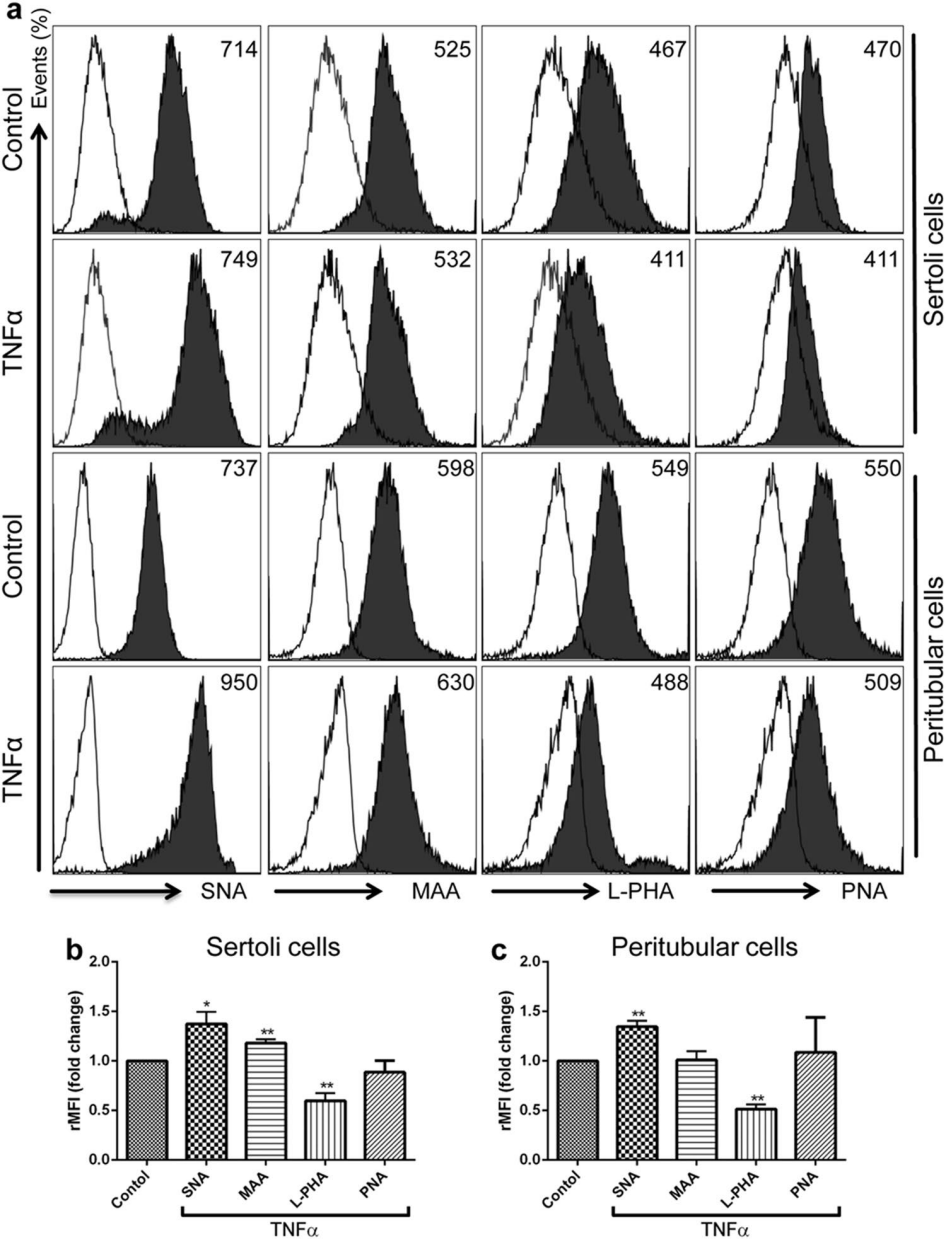
Influence of TNFα stimulation on the glycan profile of Sertoli cells and peritubular cells. (**a**) Flow cytometric analysis of cell-surface glycans in Sertoli cells and peritubular cells after stimulation with 25 ng/ml TNFα was detected by staining cells with FITC-labeled lectins (SNA, MAA, L-PHA, or PNA) (black filled histograms) or without (open histograms). Numbers in the upper-right corner represent the median of fluorescence intensity (black filled histograms). The binding of FITC-labelled lectins to Sertoli cells (**b**) or peritubular cells (**c**) was quantified as relative median fluorescence intensity (rMFI); (rMFI = (MFI with lectin – MFI without lectin)/MFI without lectin) (n = 3–5, *P < 0.05, **P < 0.01, ***P < 0.001).

Since inflammatory conditions influence the glycophenotype of cells, we investigated the binding of different lectins that selectively recognize specific oligosaccharide structures to TNFα stimulated primary Sertoli cells and peritubular cells by flow cytometry (Fig. 4a). The binding of SNA to Sertoli (Fig. 4b) and peritubular cells (Fig. 4c) challenged by TNFα was significantly increased compared to untreated cells. In contrast, the binding of L-PHA to TNFα stimulated Sertoli cells (Fig. 4b) and peritubular cells (Fig. 4c) was significantly reduced as compared to control cells. Increased binding of MAA was only found in TNFα stimulated Sertoli cells, whereas the binding of PNA to stimulated Sertoli and peritubular cells was unchanged (Fig. 4).

### Gal-1 is upregulated in Sertoli cells after TNFα stimulation

**Figure 5:**
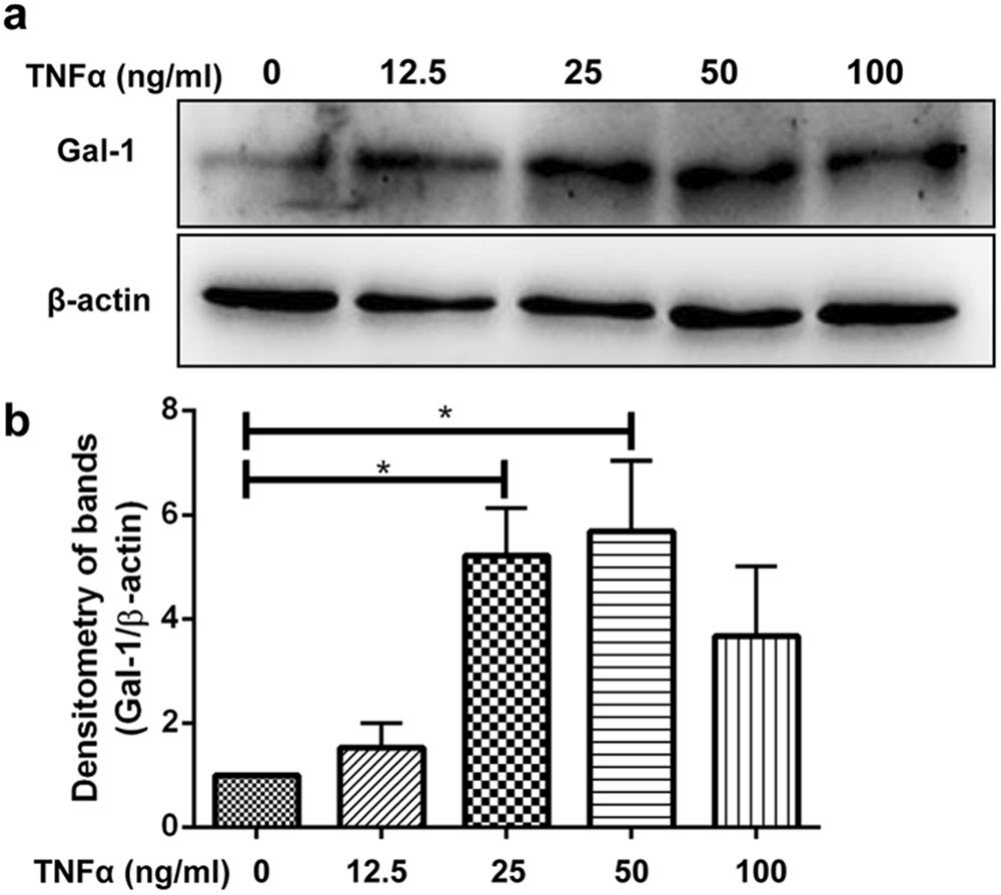
Analysis of Gal-1 expression in primary Sertoli cells. Western blot (**a**) and densitometric (**b**) analysis of Gal-1 expression in primary Sertoli cells after TNFα stimulation. The blots were cropped and entire/uncut blots are presented in the supplementary data (Supplementary Fig. 3) (n = 5, *P < 0.05).

The inflammatory cytokine TNFα is highly upregulated in EAO testis and is involved in testicular damage^11,32^. To examine the influence of an inflammatory environment on Gal-1 expression, TNFα was used to stimulate primary Sertoli cells. After stimulation a dose-dependent increase in Gal-1 expression was observed in Sertoli cells as compared to untreated cells (Fig. 5 and Supplementary Fig. 3).

### Gal-1 and TNFα synergistically induce an inflammatory response in Sertoli cells

**Figure 6:**
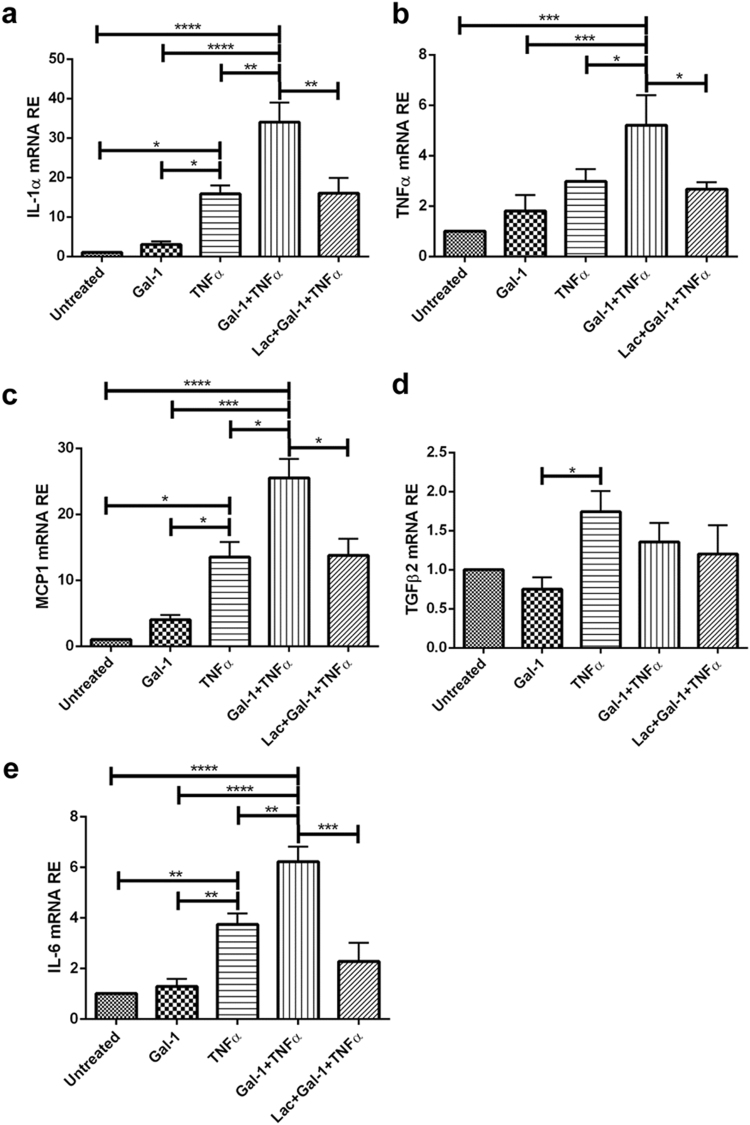
Gal-1 and TNFα act synergistically on the expression of pro-inflammatory mediators in Sertoli cells. Primary Sertoli cells were pretreated with Gal-1 (5 µg/ml) for 2 h, and then stimulated with TNFα (25 ng/ml) for 6 h. Lactose (Lac, 50 mM) was used as an inhibitor of Gal-1 binding. Relative mRNA expression of IL-1α (**a**), TNFα (**b**), MCP1 (**c**), TGFβ2 (**d**), and IL-6 (**e**) was normalized to Hprt; (n = 3–5, *P < 0.05, **P < 0.01, ***P < 0.001).

To determine whether binding of Gal-1 to Sertoli cells can modulate the inflammatory response, we analyzed expression of inflammatory cytokines in TNFα-stimulated (25 ng/ml) Sertoli cells and stimulated cells that were pretreated with Gal-1 (5 µg/ml). In Sertoli cells treated with TNFα only, mRNA expression of IL-1α (Fig. 6a), MCP1 (Fig. 6c) and IL-6 (Fig. 6e) was increased. In contrast, mRNA expression of TGFβ2 was not affected after TNFα stimulation as compared to untreated cells although it was increased when compared to stimulation with Gal-1 alone (Fig. 6d). Pretreatment of Sertoli cells with recombinant Gal-1 prior to the addition of TNFα synergistically induced expression of IL-1α, TNFα, MCP1, and IL-6 mRNA (Fig. 6a–c,e). These effects were abrogated by the addition of lactose to the Gal-1 solution 5 min prior to stimulation of Sertoli cells. Of note, Sertoli cells did not respond with an inflammatory response after treatment with Gal-1 alone (Fig. 6).

### Gal-1 and TNFα synergistically activate phosphorylation of MAPK p38 and JNK

**Figure 7:**
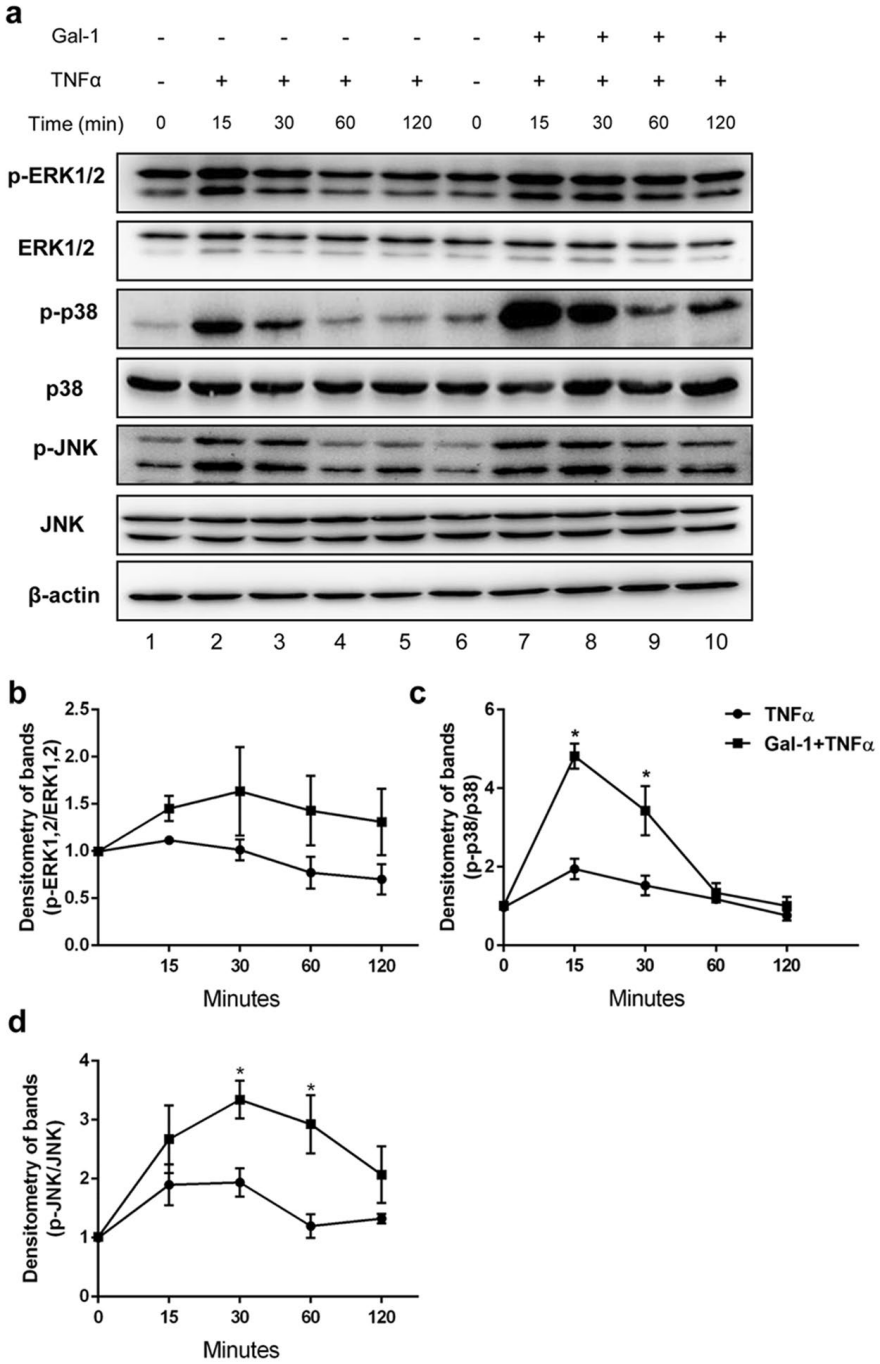
Effects of Gal-1 and TNFα on MAPK phosphorylation in Sertoli cells. (**a**) Isolated Sertoli cells were pretreated with Gal-1 (5 μg/ml; 2 h) and then stimulated with TNFα (25 ng/ml) for 0–120 min. Subsequently, phosphorylation of MAP kinases ERK1/2, p38 and JNK was investigated by Western blotting. Blots are representative of at least three independent experiments. The blots were cropped and the full-length blots are presented in Supplementary Fig. 4. Densitometric analyses of Fig. 7a are shown in Fig. 7b,c; (n = 3, *P < 0.05).

**Figure 8:**
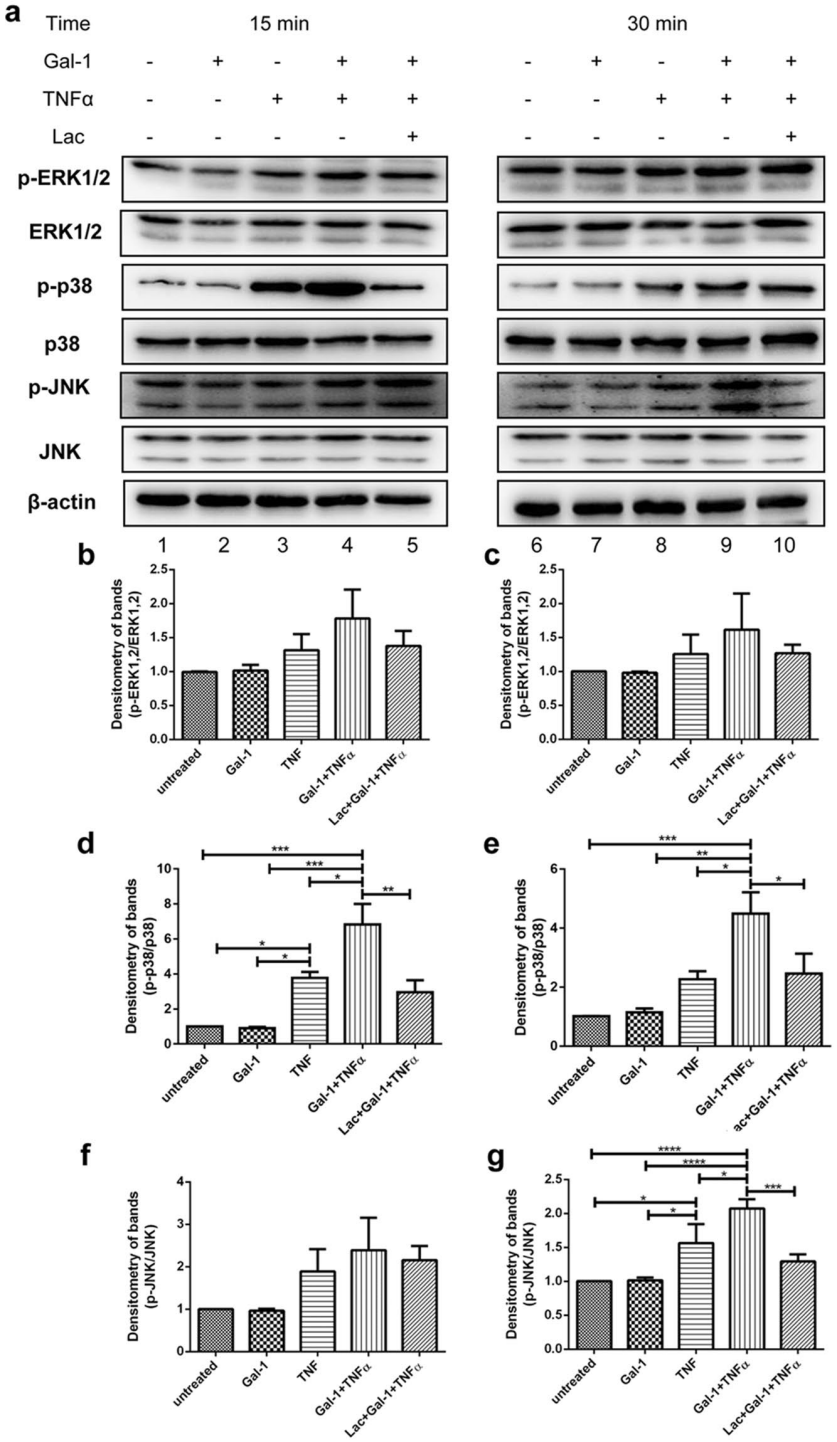
Lactose abrogates effects of Gal-1 on MAPK phosphorylation in TNFα treated Sertoli cells. (**a**) Gal-1 was pre-incubated with lactose (50 mM) for 5 min prior to addition to Sertoli cells. After 2 h Sertoli cells were stimulated with TNFα (25 ng/ml) for the indicated times. Subsequently phosphorylation of MAP kinases ERK1/2, p38 and JNK was investigated by Western blotting. Blots are representative of at least three independent experiments. The blots were cropped and the entire/uncut blots are presented in the Supplementary Fig. 6. Densitometric analyses of Fig. 8a are shown in Fig. 8b–g. Stimulation with TNFα was for 15 min (**b**,**d**,**f**) or 30 min (**c**,**e**,**g**); (n = 3–4, *P < 0.05, **P < 0.01, ***P < 0.001, ****P < 0.0001).

To better understand the mechanisms underlying the synergistic effects of Gal-1 and TNFα on the expression of pro-inflammatory cytokines IL-1α and MCP1, we evaluated the activation kinetics of mitogen-activated protein kinases (MAPK) in Sertoli cells following TNFα and Gal-1 treatment. Sertoli cells stimulated with TNFα showed increased phosphorylation of p38 and JNK from 15–30 min after stimulation (Fig. 7a, lanes 2 and 3). Interestingly, pretreatment of Sertoli cells with Gal-1 prior to TNFα stimulation synergistically enhanced phosphorylation of p38 and JNK 15–30 min after stimulation (Fig. 7a, compare lanes 2 and 3 with lanes 7 and 8; Supplementary Fig. 4; Fig. 7c,d). In contrast, no activation of MAPK was detected when Sertoli cells were treated with Gal-1 alone (Fig. 8a, compare lanes 1 and 2, and 6 and 7, Fig. 8b–g and Supplementary Figs 5 and 6). Gal-1 induced phosphorylation of p38 and JNK in the presence of TNFα was specific, because the effect could be abrogated by adding 50 mM lactose (Fig. 8a, lanes 5 and 10, Fig. 8d,e,g; Supplementary Fig. 6).

### Treatment of Sertoli cells with p38 and JNK inhibitors abrogates Gal-1 and TNFα induced expression of IL-1 α, TNFα, IL-6 and MCP1 mRNA

**Figure 9:**
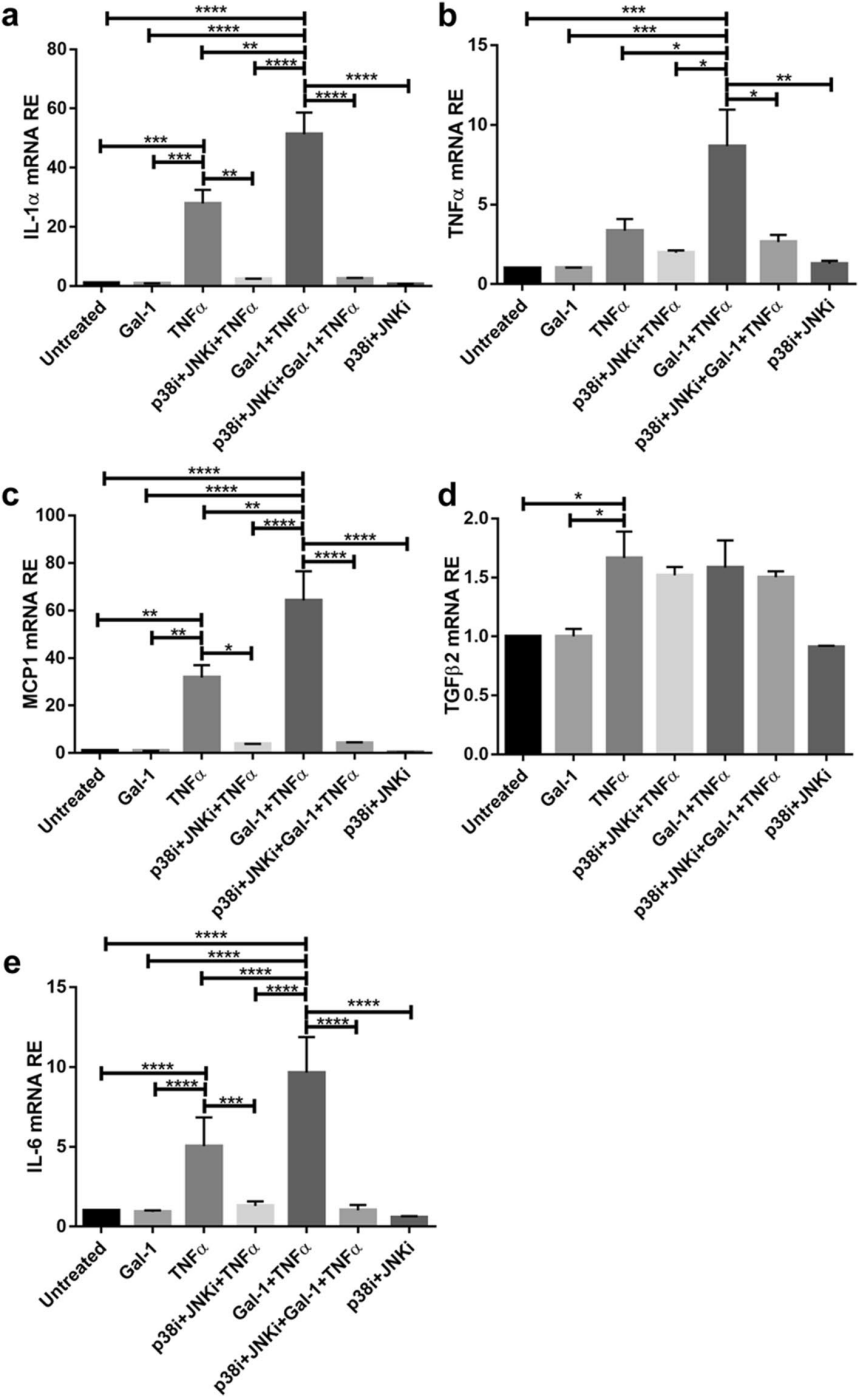
Inhibitors of p38 and JNK MAP kinases reverse the synergistic effect of Gal-1 and TNFα on expression of inflammatory mediators in Sertoli cells. Primary Sertoli cells were pretreated with Gal-1 (5 µg/ml) for 1 h prior to addition of p38 inhibitor SB 203580 (5 mM; p38i) and JNK inhibitor SP600125 (20 mM; JNKi) for 1 h. Afterwards Sertoli cells were stimulated with TNFα (25 ng/ml) for 6 h. Relative mRNA expression of IL–1α (**a**), TNFα (**b**), MCP1 (**c**), TGFβ2 (**d**) and IL-6 (**e**) was normalized to Hprt; (n = 4–7, *P < 0.05, **P < 0.01, ***P < 0.001, ****P < 0.0001).

In order to determine whether the effect of Gal-1 and TNFα on the inflammatory cytokine response is induced specifically through MAPK signaling, we used a p38 inhibitor (SB 203580, 5 mM) and a JNK inhibitor (SP600125, 20 mM) during stimulation with Gal-1 and TNFα. In the presence of both inhibitors Gal-1 and TNFα stimulated mRNA expression of IL-1α (Fig. 9a), TNFα (Fig. 9b), MCP1 (Fig. 9c) and IL-6 (Fig. 9e) was completely abrogated.

Treatment of Sertoli cells with either p38 inhibitor alone (Supplementary Fig. 7) or JNK inhibitor alone (Supplementary Fig. 8) considerably reversed synergistic expression of IL-1α, TNFα, MCP1 and IL-6 mRNA after induction with Gal-1 and TNFα.

## Discussion

An increasing body of evidence indicates that Gal-1 has immunoregulatory functions in autoimmune disease models including systemic lupus erythematosus^33^, lysolecithin-induced demyelination^34^ and experimental autoimmune encephalomyelitis (EAE)^35^ through mechanisms like selective induction of Th1 and Th17 cell apoptosis, inhibition of T cell trafficking, expansion of tolerogenic dendritic cells and regulatory T cells. Taking into account that Gal-1 is also abundantly expressed in immune privileged organs such as the placenta, ovary and testis^28^, we propose that it could play a role in the maintenance of immune privilege and conversely inflammation. We therefore investigated the expression of Gal-1 and relevant glycans in the inflamed rat testis and asked whether Gal-1 can modulate the pro-inflammatory response of Sertoli cells challenged with TNFα.

In line with previous studies^29–31,36^, we also found that Gal-1 is mainly expressed in Sertoli cells and germ cells of the normal rat testis and shows a stage-specific expression pattern. In the EAO testis, however, some CD68+ macrophages in the vicinity of granulomas were positive for Gal-1 too. Since 60–70% of macrophages in granuloma lesions are CD68 positive and local macrophage proliferation plays a key role in granuloma formation^37^, it is possible that Gal-1 induces macrophage proliferation and promotes granuloma formation. This is supported by Kanda *et al*. who showed that Gal-1 can induce proliferation of vascular endothelial cells^38^. Several studies have also shown that Gal-1 could be involved in the resolution of inflammation by regulating inflammatory signaling as well as accumulation and phagocytosis in macrophages and microglial cells^39–41^. Similar to the inflamed testis, Gal-1 expression was limited to macrophages in the vicinity of spinal cord lesions^39^. Moreover, Gal-1 was preferentially expressed by peritoneal CD11b^high^ macrophages as compared to CD11b^low^ macrophages. The CD11b^high^ macrophages had a distinct phenotype characterized by a decreased expression of TNFα and IL-1β, and increased expression of TGFβ. Expression of Gal-1 declined once the cells were converted to the CD11b^low^ phenotype as shown in a mouse peritonitis model^40^. It can be speculated that Gal-1 facilitates the resolution of macrophage-mediated inflammation during peritonitis.

Gal-1 expression was downregulated in the inflamed testis, whereas its mRNA expression did not change when qPCR results were normalized to the Sertoli cell specific transcript Sox9. This suggests that downregulation of Gal-1 in the EAO testis is the consequence of germ cell loss. These results are in line with data published in a mouse model of EAO^42^. However, direct *in vitro* treatment of isolated Sertoli cells with TNFα led to upregulation of Gal-1 expression and addition of exogenous Gal-1 potentiated the TNFα effect on the expression of IL-1α, IL-6, MCP1 and also TNFα itself. Gal-1 could play different roles whether it acts early or late during the course of acute or chronic inflammation. Therefore, higher Gal-1 levels induced by inflammatory mediators in the inflamed testis could play a pro-inflammatory role in contrast to an anti-inflammatory function sustaining immune privilege in the healthy testis. In the latter state Gal-1 produced by Sertoli cells in the absence of an inflammatory stimulus like TNFα is able to promote differentiation of tolerogenic dendritic cells and regulatory T cells, further supporting a role of endogenous Gal-1 in the maintenance of testicular immune privilege^43^. Of note, the number of regulatory T cells is increased during the course of EAO^44,45^ although these cells are not able to prevent tissue damage.

As shown by Wang *et al*. the glycocalyx signature of cells is changed in autoimmune diseases like multiple sclerosis and its animal model EAE^46^. Deficiency of the N-glycan branching enzyme Mgat5 in mice promotes T cell activity, endocytosis of CTLA-4 and autoimmunity, including a spontaneous multiple sclerosis (MS)-like disease^47^. Our data show that in EAO testis terminal sialic acid and expression of the sialyltransferase St6gal1 were upregulated, while Mgat5 mRNA expression and L-PHA binding were decreased. Moreover, SNA binding to primary Sertoli cells and peritubular cells was increased after TNFα stimulation, whereas L-PHA was downregulated. Collectively, these findings suggest that under inflammatory conditions the glycan composition on the Sertoli and peritubular cell surface becomes less favorable for Gal-1 binding, a means to dampen excessive immune reactions elicited otherwise by concerted TNFα and Gal-1 action in sterile testicular inflammation. Similar to our findings, Benjamin *et al*. reported that α-2-6 sialic acid was significantly increased on the surface of adipocytes after induction of insulin resistance with TNFα^48^. Likewise, primary human umbilical vein endothelial cells showed considerable expression of L-PHA-reactive Mgat5-modified N-glycans, that decreased significantly following exposure to pro-inflammatory cytokines like IFNγ and IL-17, whereas α-2-6 sialic acid expression was increased following IFNγ/IL-17 stimulation^27^.

We could also show that Gal-1 and TNFα synergistically increased the expression of inflammatory mediators in Sertoli cells, whereas Gal-1 alone had no effect. This synergistic effect is specific because it was abrogated in the presence of lactose and is mediated through phosphorylation of MAPKs p38 and JNK. A previous study showed that binding of Gal-1 to N-glycan modified CD45 can prolong retention of CD45 on the surface of microglial cells and augment its phosphatase activity^41^. Thus, lectin-glycan interactions can control cell responses by adjusting thresholds of cellular activation.

Recent studies show that mice lacking Gal-1 developed a reduced incidence and severity of symptoms in experimental models of epileptic seizures^49^ and orchitis^42^, which indicates a proinflammatory function of Gal-1. In EAO mice deficient for Gal-1 decreased numbers of apoptotic germ cells were found compared to normal EAO mice^42^. Considering that Gal-1 was found to be strongly expressed on apical stalks of Sertoli cells during spermiation, it could be involved in the elimination of defective germ cells. This hypothesis is supported by studies showing that Gal-1 induces T cell and Leydig tumor cell apoptosis by binding to Fas^50^. Moreover, upregulation of Fas expression was found in aberrant meiotic and postmeiotic germ cells^51^. Because the number of Fas positive apoptotic germ cells was upregulated in EAO testes^52^, Gal-1 and Fas could also mediate germ cell apoptosis in orchitis.

Considering our findings that TNFα stimulates Gal-1 expression in Sertoli cells and the addition of exogenous Gal-1 enhances pro-inflammatory effects of TNFα, we propose that under normal conditions Gal-1 induces apoptosis of defective germ cells, whereas under inflammatory conditions Gal-1 adopts a pro-inflammatory and pro-apoptotic function involved in the induction of immune responses and germ cell sloughing.

In conclusion, our data show that Gal-1 and changes in the cellular glycocalyx are involved in the regulation of immune responses in the testis. Under inflammatory conditions Gal-1 specifically enhances TNFα-stimulated production of pro-inflammatory mediators in Sertoli cells. Thus, targeting the Gal-1-glycan axis may offer a new therapeutic strategy for the treatment of autoimmune based testicular inflammation.

## Materials and Methods

### Induction of EAO

**Figure 10:**
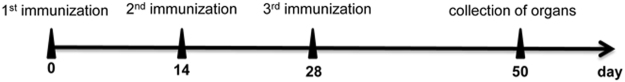
Schematic diagram illustrating induction of EAO in Wistar rats by active immunization with testicular homogenate (TH) in complete Freund’s adjuvant followed by *i*.*v*. injection of inactivated *Bordetella pertussis* bacteria. Control adjuvant animals received saline instead of TH.

Adult male Wistar rats (Charles River Laboratories, Sulzfeld, Germany) aged 60–70 days were fed with standard food pellets and water ad libitum. EAO was induced by immunization with testicular homogenate in complete Freund’s adjuvant as previously described^44^. Briefly, animals were injected with testicular homogenate in complete Freund’s adjuvant three times every 14 days (Fig. 10). Control animals received 0.9% NaCl instead of testicular homogenate in complete Freund’s adjuvant. Normal untreated rats were also included. Animals were sacrificed 50 days after the first immunization; testes were collected and frozen in liquid nitrogen. All animal experiments were approved by the local animal ethics committee (Regierungspraesidium Giessen GI 20/23 – Nr. 33/2008). All experiments involving animals were carried out in strict accordance with the recommendations in the guide for the Care and Use of Laboratory Animals of the German law of animal welfare. All methods were carried out in accordance with the approved guidelines.

### Immunofluorescence staining

Testicular cryosections (10 µm) were fixed in methanol (Sigma-Aldrich, Steinheim, Germany) at −20 °C for 10 minutes and permeabilized in 0.1% Triton X-100. After blocking with 5% goat serum in 2.5% BSA (Carl Roth, Karlsruhe, Germany), sections were incubated overnight with appropriate primary antibody at 4C (Table 1). Sections were washed in PBS and incubated with secondary antibody at room temperature (RT) for 1 hour. Finally, sections were mounted with ProLong Gold Antifade Mountant with DAPI (Thermo Fisher Scientific, Carlsbad, USA). Images were taken with an Axioplan 2 microscope (Carl Zeiss, Jena, Germany).

**Table 1:** List of antibodies and antibody dilutions used (*Western blotting, **Immuno-fluorescence).

Primary antibodies	Manufacturer	Catalogue No.	Dilution
Monoclonal rabbit anti galectin-1	GeneTex, USA	GTX62666	1:400*/1:200**
Monoclonal mouse anti rat CD68	AbD Serotec, UK	MCA341R	1:200**
Monoclonal mouse anti rat CD163	AbD Serotec, UK	MCA342R	1:200**
Monoclonal mouse anti α-smooth muscle actin	Sigma-Aldrich, Germany	F3777	1:1000*
Monoclonal mouse anti-β-actin	Sigma-Aldrich, Germany	A5441	1:10000*
Polyclonal mouse anti rat vimentin	Sigma-Aldrich, Germany	V6630	1:200*
Polyclonal rabbit anti rat phospho-p38 MAPK (Thr180/Tyr182)	Cell Signaling Technology, Germany	9211	1:1000*
Polyclonal rabbit anti rat p38 MAPK	Cell Signaling Technology, Germany	9212	1:1000*
Polyclonal rabbit anti rat phospho-p44/42 MAPK (ERK1/2) (Thr202/Tyr204)	Cell Signaling Technology, Germany	9101	1:1000*
Polyclonal rabbit anti rat p44/42 MAPK (ERK1/2)	Cell Signaling Technology, Germany	9102	1:1000*
Phospho-SAPK/JNK (Thr183/Tyr185) Antibody	Cell Signaling Technology, Germany	9251	1:1000*
SAPK/JNK Antibody	Cell Signaling Technology, Germany	9252	1:1000*
**Secondary antibodies**	**Manufacturer**	**Catalogue No**.	**Dilution**
Goat anti-Rabbit IgG-Alexa Fluor 546	Thermo Fisher Scientific, USA	A11071	1:1000**
Goat anti-mouse IgG-Alexa Fluor 448	Thermo Fisher Scientific, USA	A10684	1:1000**
Goat anti rabbit IgG-HRP	ICN, USA	55676	1:10000*
Sheep anti mouse IgG-HRP	Sigma-Aldrich, Germany	A5906	1:10000*

### Lectin binding assay

Testicular cryosections were fixed in 2% paraformaldehyde for 30 min. After blocking with 1% BSA in PBS, sections were incubated with a specific lectin (Table 2) conjugated with FITC (EY Laboratories, San Mateo, USA) in 1% BSA-PBS for 30 min. Finally, sections were mounted with ProLong Gold Antifade Mountant with DAPI. Images were taken with an Axioplan 2 microscope (Carl Zeiss, Jena, Germany).

**Table 2:** List of lectins used in this study.

Lectin	Origin	Carbohydrate specificity	Final concentration
MAA, FITC conjugated	*Maackia amurensis*	sialic acid α-2-3 galactose	20 ug/ml
PNA, FITC conjugated	*Arachis hypogaea*	terminal β-galactose	20 ug/ml
SNA, FITC conjugated	*Sambucus nigra*	sialic acid attached to terminal galactose in an α-2-6 linkage	10 ug/ml
L-PHA, FITC conjugated	*Phaseolus vulgaris*	β-1-6 branching on complex N-glycans	20 ug/ml

### Expression and purification of recombinant Gal-1

A pET-21a (+) vector for bacterial expression of human wild type Gal-1 was generously provided by Dr. Ken-Ichi Kasai and Dr. Jun Hirabayashi (Research Center for Medical Glycoscience, AIST, Tsukuba, Japan). The plasmid was amplified in *E*. *coli* DH5α and subsequently transformed into *E*. *coli* BL21 (DE3) pLysS for expression of the protein. The resulting protein was purified by affinity chromatography on an asialofetuin Sepharose column^53^. Purified Gal-1 was dialyzed three times against 100 mM NaHCO_3_ (pH 6–9) for 2 hours each and subjected to Triton X-114 (Sigma-Aldrich, Steinheim, Germany) extraction for the removal of contaminating endotoxins^54^. The protein concentration was measured by using the Pierce BCA Protein Assay Kit (Thermo Fisher, Carlsbad, USA) according to the user’s manual.

### Isolation and *in vitro* treatment of testicular cells

Peritubular and Sertoli cells were isolated from 19-day-old Wistar rats (Charles River Laboratories, Sulzfeld, Germany) using enzymatic digestion as described previously^55^. Briefly, the decapsulated testes were digested in trypsin-DNase I solution for 4–6 min. After washing, the seminiferous tubules were incubated with a collagenase-hyaluronidase-DNase I solution for 10–12 min. The supernatant containing peritubular cells was harvested, and the remaining seminiferous tubules were digested with hyaluronidase-DNase I solution. After filtration through 70 µm nylon mesh, Sertoli cells were seeded onto 6-well plates using serum free RPMI-1640 medium. Following 2 days of culture, contaminating germ cells were removed by hypotonic shock (25 mM Tris buffer, pH 7.5, 1.5 min). Purity of isolated Sertoli and peritubular cells was confirmed to be >95% by using α-smooth muscle actin (for peritubular cells) and vimentin (for Sertoli cells) immunolabeling. For flow cytometric analysis, Sertoli cells and peritubular cells were treated with 25 ng/ml TNFα (PromoCell GmbH, Heidelberg, Germany) for 48 hours.

### Flow cytometry

A total of 2 × 10^5^ Sertoli or peritubular cells were used for the lectin binding assay. After washing, cells were incubated for 1 h at RT with 50 µl of the corresponding plant lectin solution (Table 2). In order to exclude dead cells propidium iodide was added before the measurement and samples were analyzed with a MACSQuant 10 flow cytometer (Miltenyi Biotec, Bergisch Gladbach, Germany). Data were collected from 20,000 events and analyzed with FlowJo soſtware version 10.0.8 (Ashland, Oregon, USA).

### Western blotting

Testes or Sertoli cells were homogenized in ice-cold RIPA buffer (50 mM Tris-HCl pH 8.0, 1% Nonidet P 40, 0.5% deoxycholate, 0.1% SDS, 150 mM NaCl) containing protease inhibitor cocktail 1 (Sigma-Aldrich, Steinheim, Germany) and Halt Phosphatase Inhibitor Cocktail (Thermo Fisher, Carlsbad, USA). Equal amounts of proteins (20 µg) were separated by SDS-polyacrylamide gel electrophoresis in 15% polyacrylamide gels and electroblotted onto nitrocellulose membranes (GE Healthcare, Freiburg, Germany). The efficiency of the transfer was monitored by Ponceau S staining. Membranes were blocked with 5% non-fat dry milk in TBS containing 0.1% Tween 20 for 1 h at RT. Blots were probed overnight with primary antibody (Table 1). Afterwards, the membranes were washed and incubated with the appropriate secondary HRP-conjugated antibody (Table 1). Signals were visualized using ECL (Millipore Corporation, Billerica, USA) and analyzed by densitometry (Fusion FX, Witec Ag, Luzern, Switzerland) to select and determine the background subtracted density of the bands in all blots. Equal loading was confirmed using β-actin and total protein signals were confirmed as controls for phosphoprotein signals.

### RNA extraction and quantitative RT–PCR

Total RNA was isolated by using RNeasy Mini kit (Qiagen, Hilden, Germany) according to the manufacturer’s instructions. Real-time RT–PCR was performed on a CFX96 Touch thermal cycler (Bio-Rad, Munich, Germany) using the iTaq Universal SYBR Green Supermix (Bio-Rad, Munich, Germany). The primers for rat galectin-1 (Lgals1), mannoside acetylglucosaminyltransferase 5 (Mgat5), core 2 glucosaminyl (N-acetyl) transferase 1 (Gcnt1), and ST6 beta-galactoside α-2-6-sialyltransferase 1 (St6gal1) were purchased as PrimePCR SYBR Green Assays from Bio-Rad. Primer sequences and amplicon sizes are shown in Table 3. β-actin, hypoxanthine guanine phosphoribosyl transferase (Hprt) and 18 s rRNA (Rn18s) were used as housekeeping genes. Relative gene expression was calculated by using the 2^−ΔΔCt^ method^56^.

**Table 3:** List of PCR primers used. F: forward primer, R: reverse primer.

Gene	Primer (5′ → 3′)	Catalogue No.	Gene ID	Amplicon size (bp)
Lgals 1 (Gal-1)	PrimePCR SYBR Green Assay	qRnoCED0001745 (Bio-Rad)	56646	63
Mgat5	PrimePCR SYBR Green Assay	qRnoCID0053085 (Bio-Rad)	65271	116
St6gal1	PrimePCR SYBR Green Assay	qRnoCED0004461 (Bio-Rad)	25197	63
Gcnt1	PrimePCR SYBR Green Assay	qRnoCED0008631 (Bio-Rad)	64043	93
Hprt	F: TCTGTCATGTCGACCCTCAG R: CCTTTTCCAAATCTTCAGCA	/	24465	109
Actb (β-actin)	F: ATGGTGGGTATGGGTCAGAA R: GGGTCATCTTTTCACGGTTG	/	81822	232
Rn18s (18s RNA)	F: TACCACATCCAAGGAAGGCAGCA R: TGGAATTACCGCGGCTGCTGGCA	/	19791	180
Sox9	F: CTGAAGGGCTACGACTGGAC R: TACTGGTCTGCCAGCTTCCT	/	140586	140
Tnf (TNFα)	F: GCCTCTTCTCATTCCTGCTC R: CCCATTTGGGAACTTCTCCT	/	24835	101
Tgfb2 (TGFβ2)	F: CCGGAGGTGATTTCCATCTA R: GCGGACGATTCTGAAGTAGG	/	81809	201
Il6 (IL-6)	F: GCCCTTCAGGAACAGCTATG R: GTCTCCTCTCCGGACTTGTG	/	24498	119
Ccl2 (MCP-1)	QuantiTect Primer Assay	QT00183253 (Qiagen)	24770	117
Il1a (IL-1α)	QuantiTect Primer Assay	QT00183670 (Qiagen)	24493	101

### Statistical analysis

Data are shown as mean ± SEM. Comparisons of lectin binding in untreated and TNFα treated cells were performed by a two-tailed t-test. One-way ANOVA followed by Tukey’s multiple comparison post hoc test were applied when more than two groups were compared. P-values < 0.05 were considered as statistically significant. All tests were performed using GraphPad Prism 5 software (GraphPad Software, San Diego, USA).

### Data availability statement

The datasets generated during the current study are available from the corresponding author on reasonable request.

## Electronic supplementary material

Supplementary Information
