# Supplementary material for: Galectin-1 enhances TNFα-induced inflammatory responses in Sertoli cells through activation of MAPK signalling
Source: Sci Rep. 2018 Feb 27;8:3741. doi: 10.1038/s41598-018-22135-w (PMC5829165; doi:10.1038/s41598-018-22135-w)
Supplement: Supplementary file 1 — Supplementary Information [file 41598_2018_22135_MOESM1_ESM.pdf]

Galectin-1 enhances TNFα-induced inflammatory responses in Sertoli cells through activation of MAPK signalling

Tao Lei[1], Sven Moos[1], Jörg Klug[1], Ferial Aslani[1], Sudhanshu Bhushan[1], Eva Wahle[1], Suada Fröhlich[1], Andreas Meinhardt[1] and Monika Fijak[1*]

[1]Department of Anatomy and Cell Biology, Justus-Liebig University, Giessen, Germany

[*]**Corresponding author:** Dr. Monika Fijak, PhD
Department of Anatomy and Cell Biology
Justus-Liebig-University of Giessen
Aulweg 123
35385 Giessen, Germany
Phone: +49-641-9947032
Fax: +49-641-9947049
Email: monika.fijak@anatomie.med.uni-giessen.de

Running title: Gal-1 enhances inflammatory response in Sertoli cells

Key words: experimental autoimmune orchitis, testicular inflammation, galectin-1, Sertoli cells, peritubular cells

Supplementary Fig. 1. Full length blots for Fig. 2a.

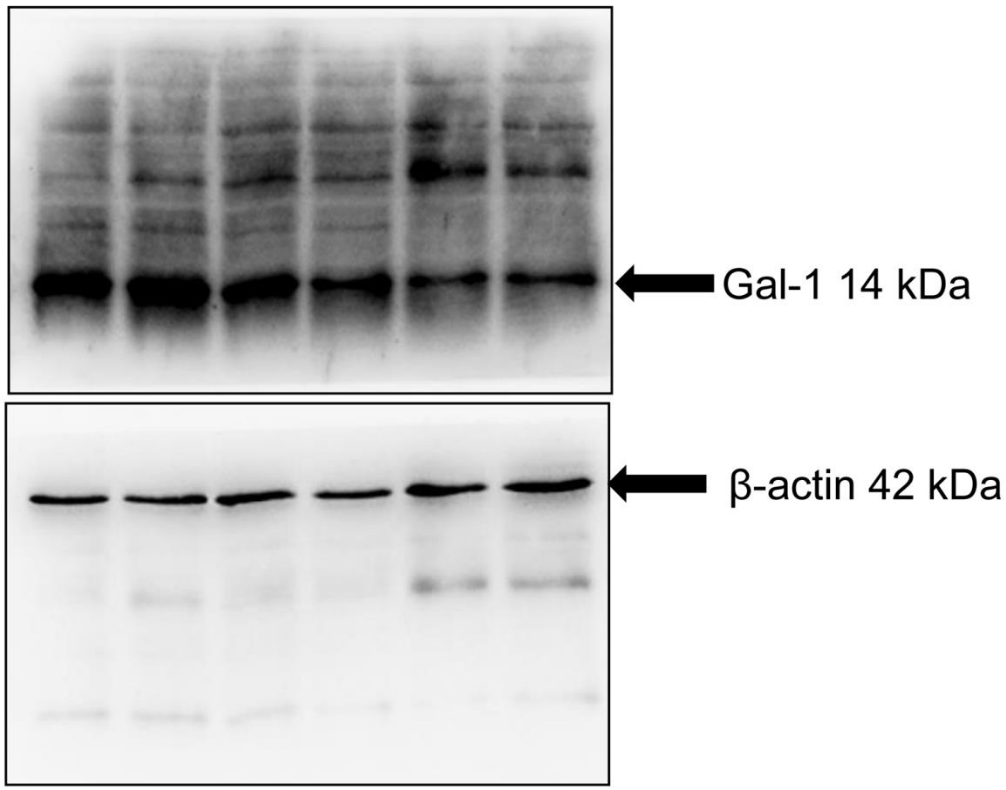

Supplementary Fig. 2.

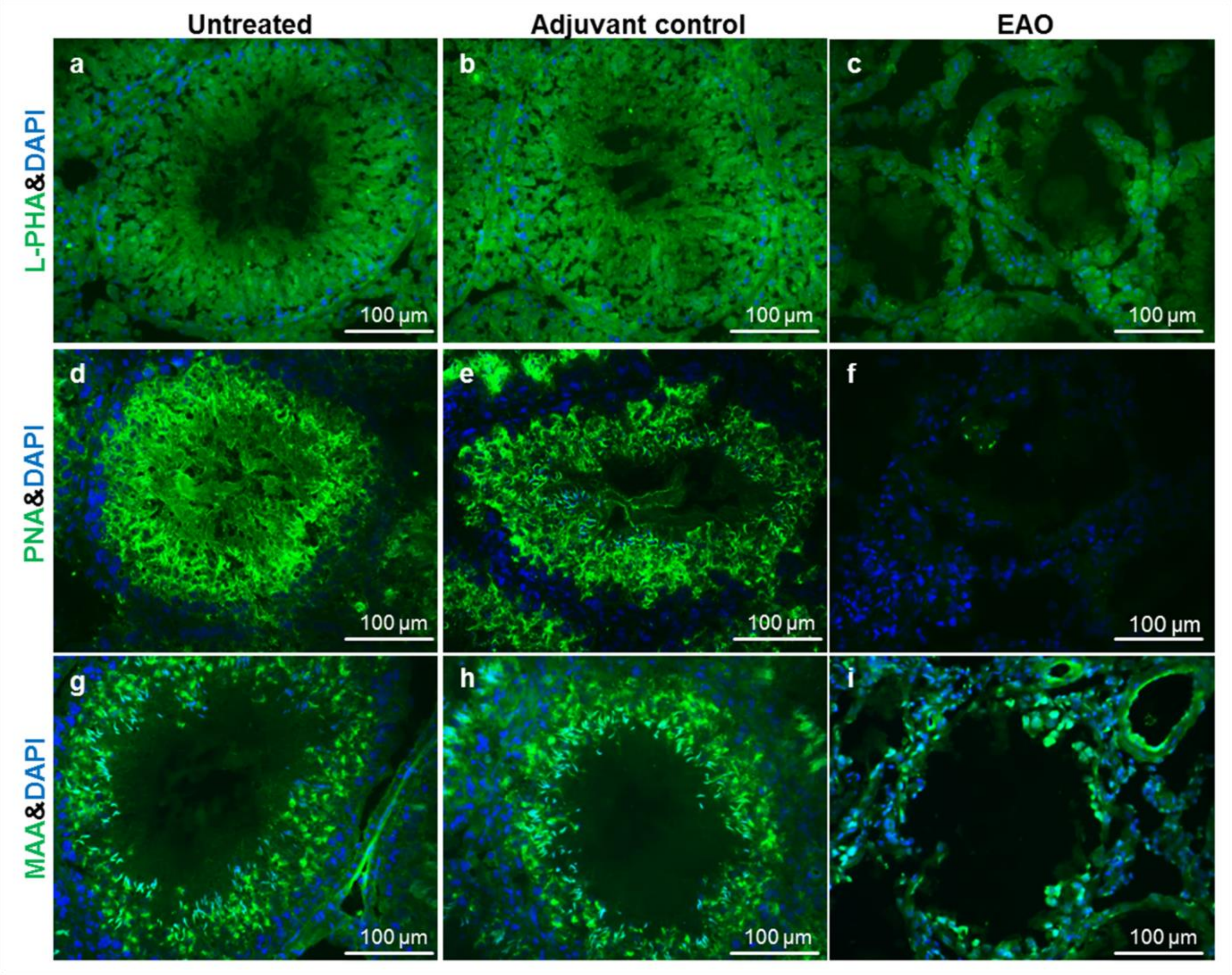

Supplementary Fig. 2. Binding of FITC labeled L-PHA (**a – c**), PNA (**d – f**), and MAA (**g – i**) to testicular sections from untreated (**a, d, g**), adjuvant control (**b, e, h**) and EAO (**c, f, i**) animals.

Supplementary Fig. 3.

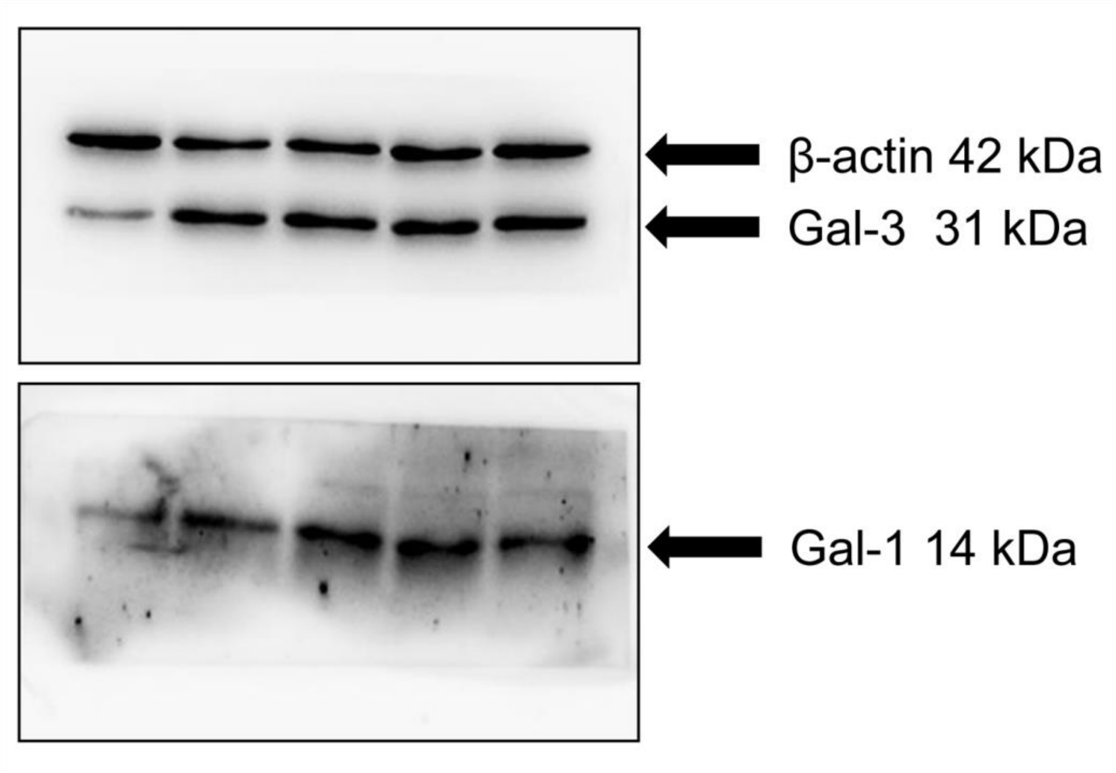

Supplementary Fig. 3. Full length blot for Fig. 5a. The membrane was cut into two pieces for the incubation with different antibodies.

Supplementary Fig. 4.

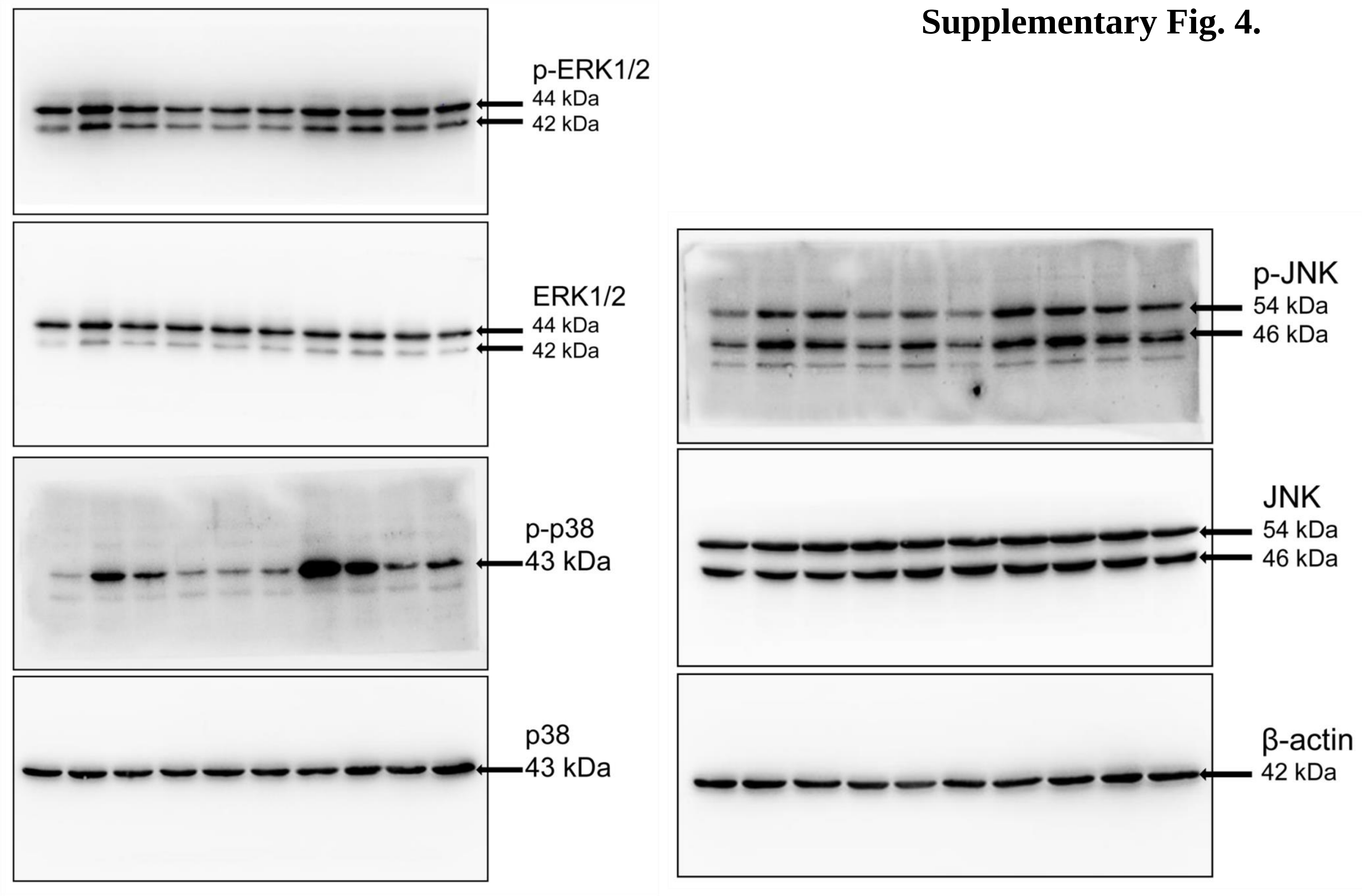

Supplementary Fig. 4. Full length blots for Fig. 7a. For quantitative analysis, total protein signals were used as controls for phosphoprotein signals.

Supplementary Fig. 5.

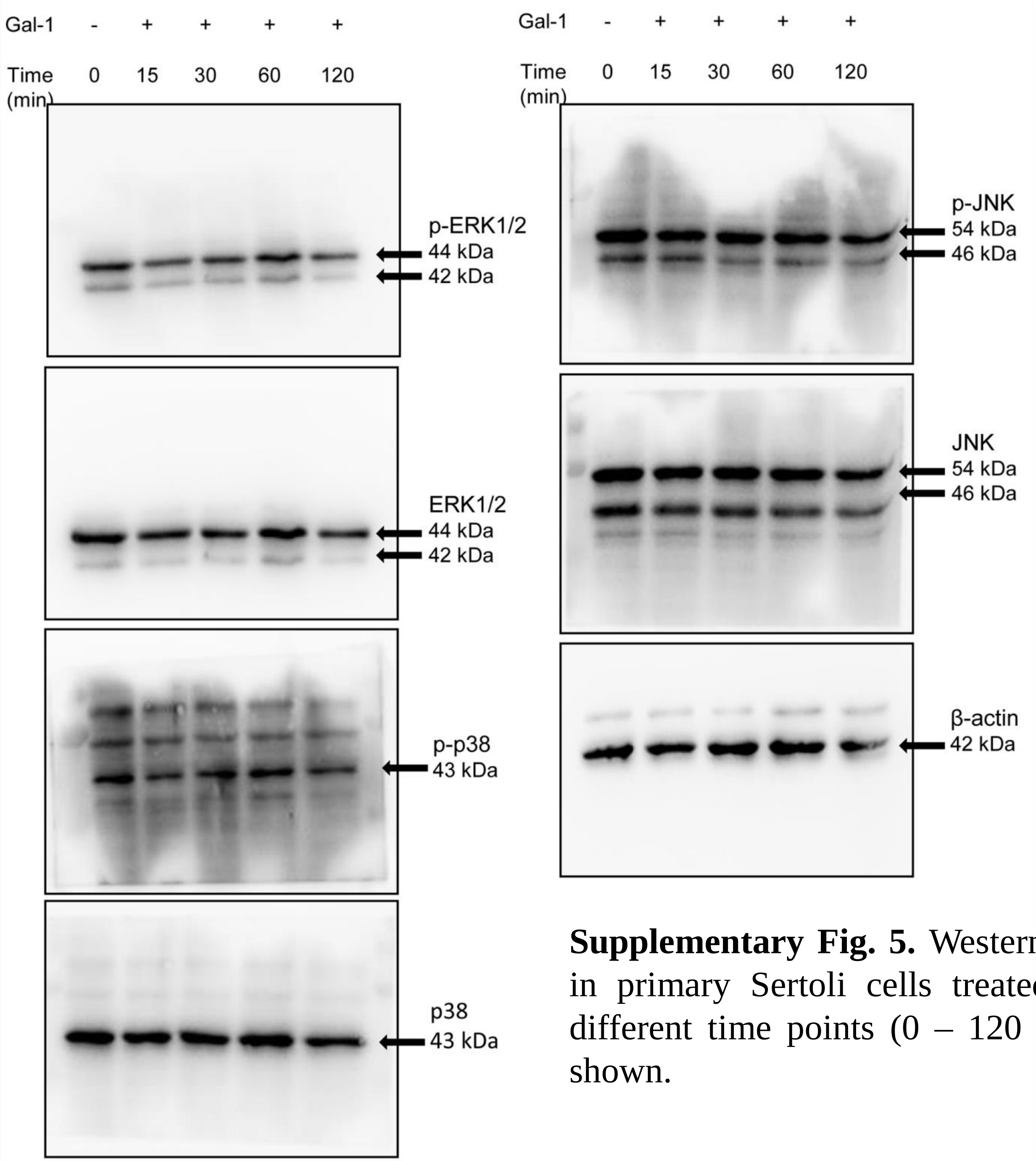

Supplementary Fig. 5. Western blot analysis of MAPK signaling in primary Sertoli cells treated with Gal-1 only (5 μg/ml) for different time points (0 – 120 min; n = 3). Full length blots are shown.

Supplementary Fig. 6.

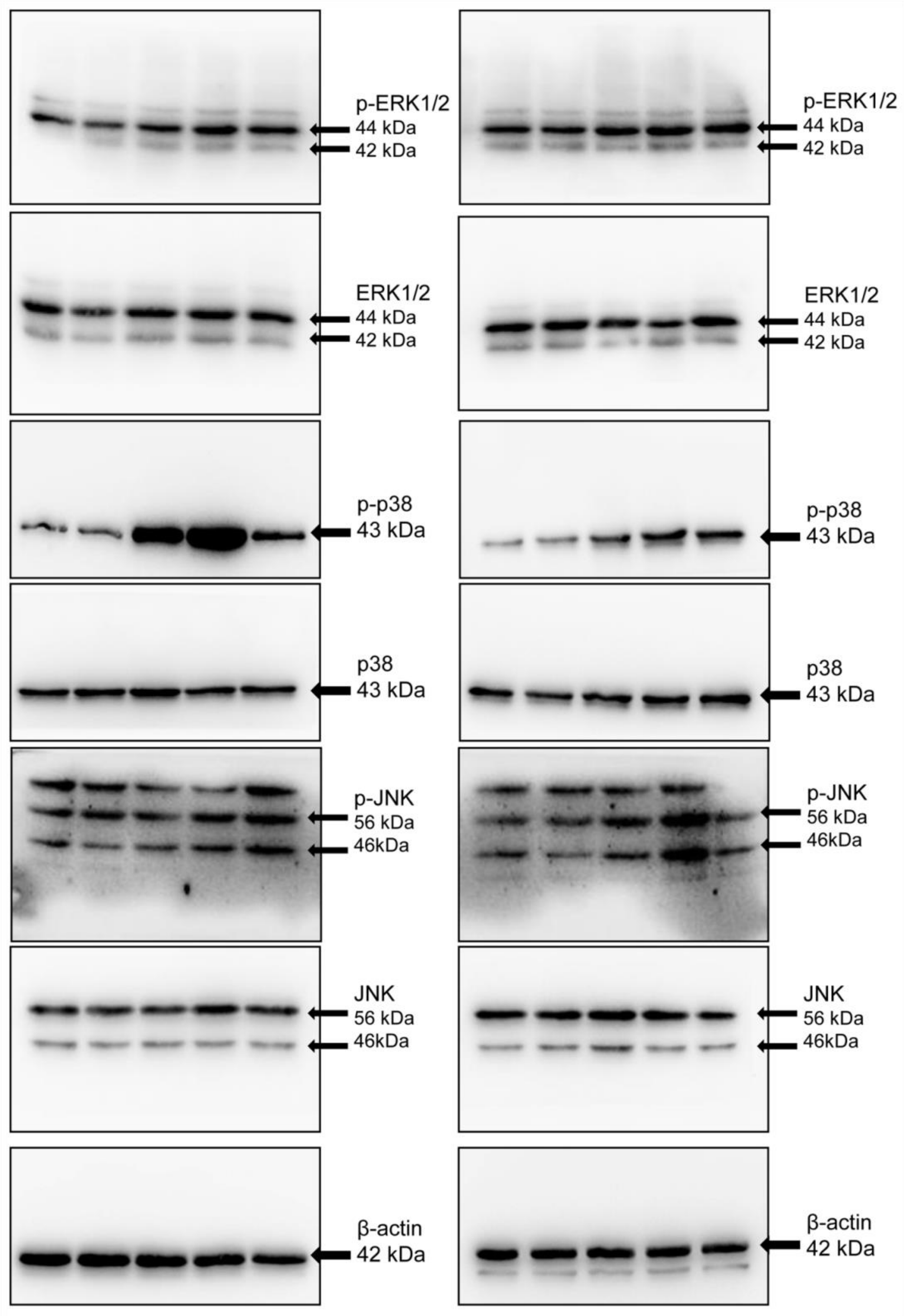

Supplementary Fig. 6. Full length blots for Fig. 8a. For quantitative analysis, total protein signals were used as controls for phosphoprotein signals.

Supplementary Fig. 7.

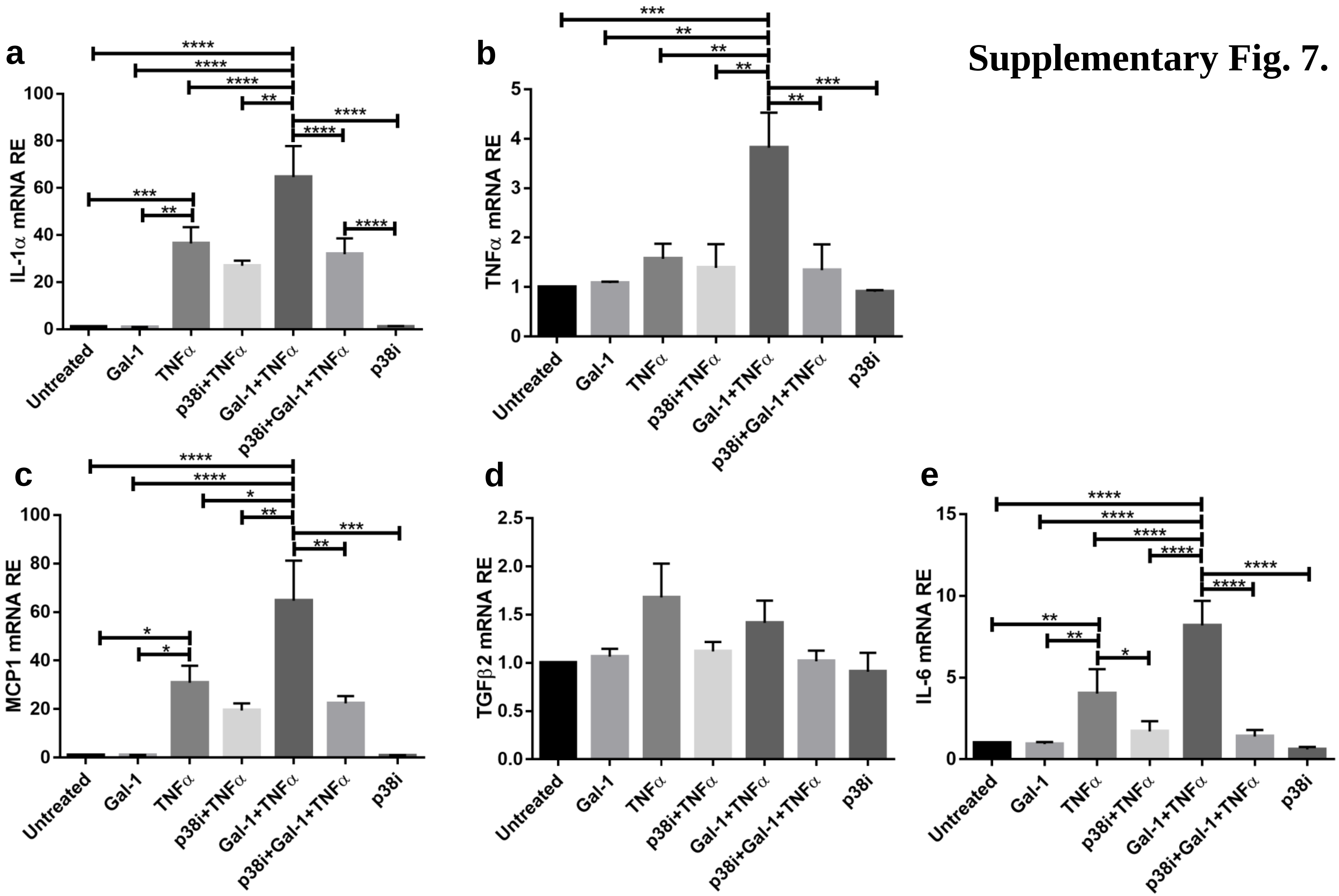

Supplementary Fig. 7. Inhibition of p38 MAP kinase partially reversed Gal-1 and TNFα induced expression of inflammatory mediators in Sertoli cells. Primary Sertoli cells were pretreated with Gal-1 (5 µg/ml) for 1 h prior to addition of p38 inhibitor SB 203580 (5 mM; p38i) for 1 h. Afterwards Sertoli cells were stimulated with TNFα (25 ng/ml) for 6 h. Relative mRNA expression of IL-1α (a), TNFα (b), MCP1 (c), TGFβ2 (d) and IL-6 (e) was normalized to Hprt; (n = 4-7, * P<0.05, ** P<0.01, *** P<0.001, **** P<0.0001).

Supplementary Fig. 8.

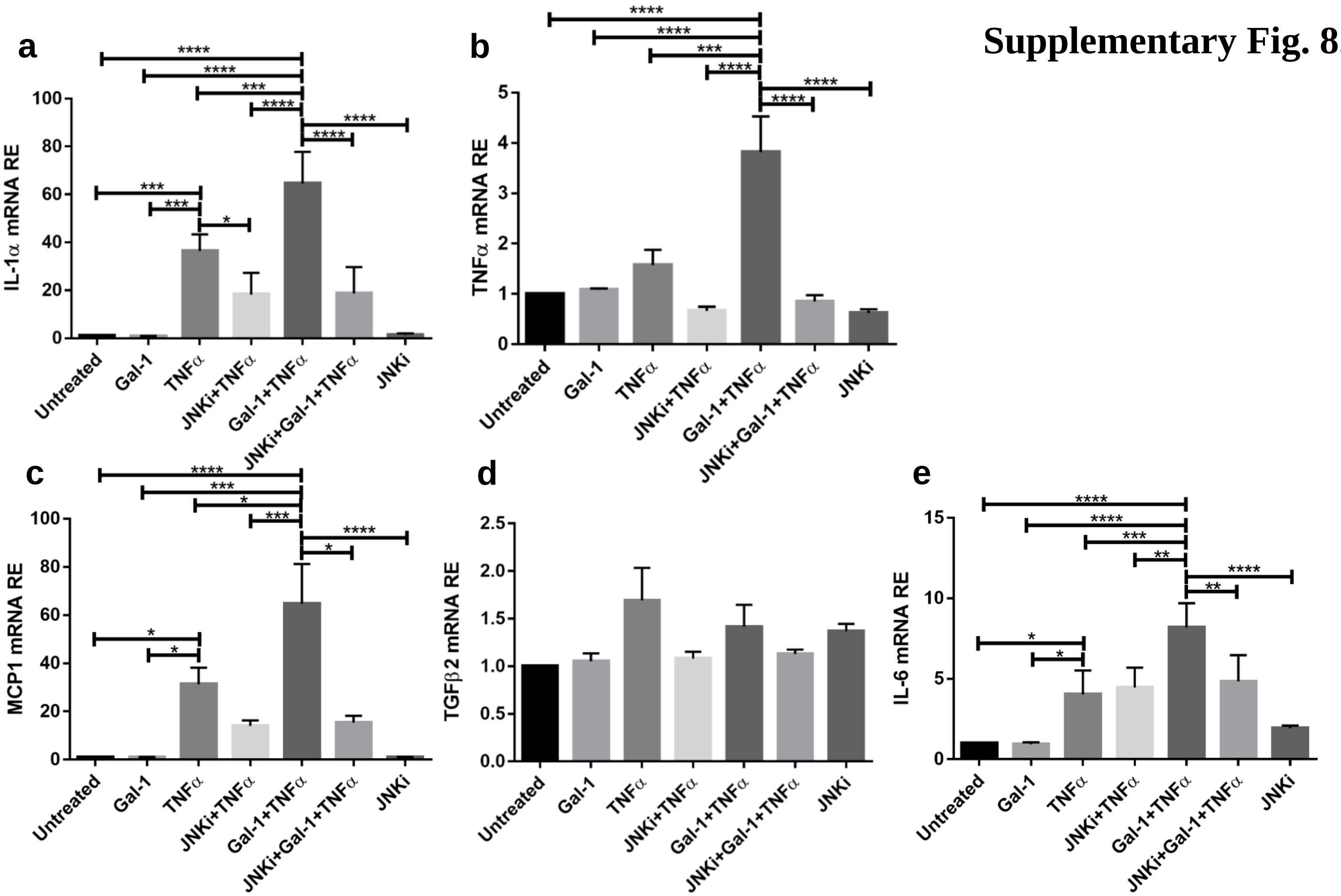

Supplementary Fig. 8. Inhibition of JNK MAP kinase partially reversed Gal-1 and TNFα induced expression of inflammatory mediators in Sertoli cells. Primary Sertoli cells were pretreated with Gal-1 (5 µg/ml) for 1 h prior to addition of JNK inhibitor SP600125 (20 mM; JNKi) for 1 h. Afterwards Sertoli cells were stimulated with TNFα (25 ng/ml) for 6 h. Relative mRNA expression of IL-1α (a), TNFα (b), MCP1 (c), TGFβ2 (d) and IL-6 (e) was normalized to Hprt; (n = 4-7, * P<0.05, ** P<0.01, *** P<0.001, **** P<0.0001).
